# TGF-β-driven NK Cells plasticity in hepatocellular carcinoma

**DOI:** 10.3389/fimmu.2025.1651129

**Published:** 2025-11-05

**Authors:** Valentina Reverberi, Anna Montali, Andrea Vecchi, Marzia Rossi, Alessio Pelagatti, Sara Doselli, Benedetta Farina, Andrea Olivani, Giorgio Economopoulos, Raffaele Dalla Valle, Diletta Laccabue, Francesca Ferraglia, Amalia Penna, Paola Fisicaro, Carolina Boni, Gabriele Missale

**Affiliations:** ^1^ Department of Medicine and Surgery, University of Parma, Parma, Italy; ^2^ Laboratory of Viral Immunopathology, Unit of Infectious Diseases and Hepatology, Azienda Ospedaliero-Universitaria of Parma, Parma, Italy

**Keywords:** NK cells, ILC1-like cells, hepatocellular carcinoma, TGF-β, immune function, functional restoration

## Abstract

**Background:**

Hepatocellular carcinoma (HCC) is a leading cause of cancer-related mortality, with limited curative options for advanced disease. Natural Killer (NK) cells are critical innate immune effectors, but their anti-tumor function is severely compromised by the immunosuppressive tumor immune microenvironment (TIME), particularly through transforming growth factor-beta (TGF-β). This study investigates the pivotal role of TGF-β signaling in modulating NK cell phenotypes and functions within the HCC TIME.

**Methods:**

to comprehensively assess TGF-β pathway activation and its impact on NK cells, tumor-infiltrating lymphocytes (TILs) and liver-infiltrating lymphocytes (LILs) were isolated from HCC patients undergoing curative resection. Phenotypic and functional analyses were performed, along with functional restoration experiments targeting TGF-β signaling.

**Results:**

Tumor-infiltrating NK cells (TINKs) exhibited significant activation of both canonical (SMAD-dependent) and non-canonical (TAK1/p38 MAPK) TGF-β signaling, with a predominance of the non-canonical pathway. This activation was associated with the emergence of an ILC1-like NK subset (CD103^+^/CD49a^+^), which was nearly absent in non-tumor liver tissue. These ILC1-like cells maintained strong cytokine production and expressed high levels of inhibitory receptors (PD-1, TIM-3, TIGIT), whereas conventional NK cells (cNKs; CD103^−^/CD49a^−^/CD9^−^) were functionally impaired. Notably, blocking TGF-β receptor binding and SMAD3 activation restored cNK functionality.

**Discussion:**

our findings suggest that while non-canonical TGF-β signaling drives phenotypic reprogramming and contributes to NK cell dysfunction, canonical SMAD-dependent signaling remains a key therapeutic target for functional restoration. These results highlight the dual role of TGF-β in immune modulation and suggest that targeted pathway inhibition could enhance innate anti-tumor responses, opening new avenues for combination therapies in HCC.

## 1 Introduction

Hepatocellular carcinoma (HCC) represents over 80% of primary liver cancers and remains one of the leading causes of cancer-related death worldwide (1). Curative treatments such as liver transplantation, surgical resection, and local ablation are effective but largely restricted to patients with early-stage disease. For those with advanced, unresectable tumors, the combination of anti–PD-L1 and anti–VEGF or anti-PD-L1 and anti-CTLA-4 are currently the standard first-line therapy. However, despite this therapeutic advancement, overall mortality in HCC remains high (2).

Natural Killer (NK) cells are critical effectors of the innate immune system and play a pivotal role in antitumor immunity. Their ability to recognize and eliminate transformed cells without prior sensitization makes them promising candidates for immunotherapeutic strategies (3). However, in HCC, the tumor immune microenvironment (TIME)—characterized by high levels of transforming growth factor-β (TGF-β), hypoxia, and nutrient deprivation—profoundly impairs NK cell function.

TGF-β represents a central regulator of immune suppression within the TIME and significantly impacts NK cell plasticity and functionality. Although its immunosuppressive effects, as well as its role in immune cells differentiation are well-established, the precise molecular mechanisms through which TGF-β modulates NK cell behavior remain incompletely understood.

*In vitro* studies have shown that TGF-β drives the conversion of peripheral NK (pNK) cells into CD49a^+^ tissue-resident-like NK cells, exhibiting a decidual-like phenotype characterized by enhanced VEGFA secretion and promotion of trophoblast invasion (4). In the context of HCC, tumor-infiltrating NK cells express higher levels of CD49a compared to their liver-resident counterparts (5), and this phenotype is associated with poor prognosis (6, 7), suggesting a potential role for CD49a^+^ NK cells in promoting immune evasion and tumor progression.

TGF-β exerts its effects via both canonical (SMAD-dependent) and non-canonical (e.g., TAK1–p38 MAPK–dependent) signaling pathways, with complex and context-dependent outcomes for NK cell function. Canonical SMAD-mediated signaling suppresses NK cell activity by downregulating activating receptors such as NKp30, NKp46, NKG2D, and DNAM-1, as well as cytolytic granules (8, 9). Several studies have highlighted the involvement of TGF-β in shaping pro-angiogenic NK populations in various cancer types (10, 11), and non-canonical signaling via the TAK1–p38 axis has been implicated in driving the conversion of NK cells toward an ILC1-like phenotype, characterized by the expression of tissue-residency markers CD103 and CD49a (12). Functionally competent tissue-resident NK cells bearing this phenotype have also been identified in human lung tumor (13, 14).

Paradoxically, despite associations with poor prognosis in HCC, tissue-resident NK cells have also been shown to synergize with conventional NK (cNK) cells in controlling liver metastases (15). Moreover, CD49a^+^ NK cells have been implicated in the orchestration of adaptive antitumor immunity, particularly through IL-12–induced enhancement of dendritic cell–CD8^+^ T cell interactions via NK cell–derived CCL5 (16).

These findings underscore the multifaceted and context-specific roles of TGF-β in shaping the NK cell landscape within tumors. While TGF-β signaling may contribute to immune evasion by dampening NK cell effector functions, it may also play a crucial role in maintaining NK cell homeostasis within the tumor microenvironment. Thus, therapeutic strategies aimed at modulating TGF-β signaling hold promise but require a nuanced understanding of the temporal and phenotypic consequences of TGF-β exposure in tumor-infiltrating NK cells.

In this study, we investigated NK cell plasticity within the TGF-β–rich HCC microenvironment, tumor enrichment of different NK-cell subsets, their function and the effect of inhibiting the different TGF-β activation pathways.

## 2 Materials and methods

### 2.1 Patients and biological samples

Tumor and non-tumorous specimens were obtained from 42 HCC patients undergoing curative resection. The diagnosis of hepatocellular carcinoma (HCC) was established based on ultrasound and confirmed with computed tomography (CT) or magnetic resonance imaging (MRI) in selected cases. All patients tested negative for anti-human immunodeficiency virus (HIV) antibodies. HCC was classified as early stage according to the Barcelona Clinic Liver Cancer (BCLC) staging system, defined as a single lesion or up to three nodules, each measuring less than 3 cm in diameter, with preserved liver function.

The study was approved by the local ethics committee (Comitato Etico Indipendente, Azienda Ospedaliero-Universitaria di Parma, Parma, Italy). All participants gave written informed consent to take part in the study.

Liver- and Tumor-infiltrating lymphocytes (LILs and TILs) were isolated following digestion of liver and tumor tissues with a resection buffer (1x PBS + 2% FCS + 2 mM EDTA) supplemented with Collagenase (1.25 mg/ml) (Sigma) and DNase (0.01 mg/ml) (Sigma) at 37 °C for 45 minutes under agitation. After digestion the supernatant was kept and separated by density gradient (Ficoll) centrifugation at 2200 rpm for 20 minutes to collect the ring of LILs and TILs. The cells were counted, frozen in freezing medium (90% fetal bovine serum and 10% dimethyl sulfoxide, DMSO) and cryopreserved in liquid nitrogen until use. The cell counts typically yielded between 10^6^ and 3-4×10^6^ total cells, maintaining post-isolation viability at no less than 85-90%. After thawing the median percentage of live cells was 83.33%, with an Interquartile Range (IQR) of 12.99%.

### 2.2 Gene set enrichment analysis

Targeted GSEA was performed on 25 curated gene sets associated with both canonical and non-canonical TGF-β signaling pathways (**Supplementary Table 1**). Gene expression data for Liver- and Tumor-infiltrating NK Cells (LINKs and TINKs), previously published, are available at the National Center for Biotechnology Information Gene Expression Omnibus (GSE183349) (17).

The analysis was conducted using the “gene set” permutation type, with all other parameters set to default. Significantly enriched gene sets were identified based on a false discovery rate (FDR) threshold of <0.25, employing 1,000 permutations and Signal2Noise statistics.

### 2.3 Phenotypic analysis of liver- and tumor-infiltrating NK-cells

For all flow cytometry analyses gating was performed on the lymphocyte population. Dead cells were eliminated using a live/dead viability dye. Natural killer (NK) cells were identified as CD3^−^CD56^+^ within the gated population and differentiation into CD56^bright^ and CD56^dim^ subpopulations was performed based on distinct CD56 expression intensities. Flow cytometric cell count revealed a median of 1.44x10^5^ total live cells and 2.46x10^4^ live NK cells.

For further phenotypic analyses and identification of infiltrating NK cells subpopulations, LINKs and TINKs were thawed, resuspended in RPMI-1640 containing 8% human serum and stained with monoclonal antibodies specific for CD3-BUV805 (BD), CD56-BUV395 (BD), CD103-BV421(BD), CD49a-BV510 (BD), CD9-PECF594 (BD), PD1-PE-Cy7 (BD), TIM3-BV786 (BD), TIGIT-PerCP-eFluor710 (Invitrogen). Stained samples were acquired with a LSR FORTESSA BD Flow Cytometer and analyzed with FACS-DIVA and FlowJo Software.

To explore potential NK cell subclusters within liver and tumor samples, a t-distributed stochastic neighbor embedding (t-SNE) analysis was performed on the concatenated flow cytometry data. t-SNE was applied to the multidimensional dataset to visualize the distribution of NK cell subsets based on marker expression. The analysis was conducted using FlowJo’s built-in t-SNE plugin with standard parameters (perplexity, learning rate and iterations), allowing unsupervised identification of phenotypically distinct NK cell populations.

### 2.4 TGF-β pathway activation

After thawing, liver- and tumor-infiltrating NK cells were stained with surface antibodies: anti-CD3-BUV805, anti-CD56-BUV395, anti-CD49a-BV510, anti-CD9-PECF594, anti-CD103-BV421, anti-PD1-PE-Cy7, anti-TIM3-BV786, anti-TIGIT-BV650 (BD). For intracellular staining Phosflow Fix Buffer I and Phosflow Buffer III (BD) were used according to manufacturer’s instructions. To assess activation of TGF-β signaling pathways, phospho-specific antibodies against Smad2 (pS465/pS467)/Smad3 (pS423/pS425) conjugated to PE (BD) and against TAK1 (pThr187) unconjugated were employed. For TAK1 detection a FITC-conjugated anti-rabbit IgG secondary antibody (BD) was used. Stained samples were acquired with a LSR FORTESSA BD Flow Cytometer and analyzed with FACS-DIVA and FlowJo Software.

### 2.5 TGF-βR1 expression in liver- and tumor-infiltrating NK cells

After thawing, LINKs and TINKs were first stained with a live/dead viability dye to exclude non-viable cells. Surface staining was performed using: anti-CD3-BUV805, anti-CD56-APC-R700, anti-CD16-FITC, anti-CD103-BV421, anti-CD49a-RB705, and anti-CD9-BUV395 (BD Biosciences).

Following surface staining, cells were washed twice with PBS 2% FBS, then fixed and permeabilized using Cytofix/Cytoperm™ Fixation/Permeabilization Kit (BD) and incubated with an unconjugated anti-TGF-βR1 antibody (R&D Systems/Bio-Techne) in Perm/Wash buffer for 30 min at 4 °C in the dark. For TGF-βR1 detection, an APC-conjugated anti-goat IgG secondary antibody (R&D Systems/Bio-Techne) was used. To ensure signal specificity, a Normal Goat IgG isotype control (R&D Systems/Bio-Techne) was included. Data were expressed after subtracting the percentage obtained with the isotype control (never exceeding 0.5–0.6%). After staining, cells were washed twice with BD Perm/Wash buffer, resuspended and acquired on LSR FORTESSA BD Flow Cytometer. Data collected were analyzed with FACS-DIVA and FlowJo Software.

### 2.6 Functional analysis of infiltrating NK-cells

IFN-γ, TNF-α production and CD107a expression of infiltrating NK-cells were evaluated after overnight stimulation with IL-12 and IL-18 (5ng/ml each). At the time of stimulation antibody anti-CD107a-PE-Cy7 was added to each sample. After 30 minutes GolgiStop and GolgiPlug (BD), protein transport inhibitors, were added to the cells. The next day surface staining with anti-CD3-BUV805, anti-CD56-BUV395, anti-CD49a-BV510, anti-CD9-PECF594, anti-CD103-BV421, anti-PD1-PE, anti-TIM3-BV786, anti-TIGIT-PerCP-Cy5.5 (BD) was performed. Cells were then fixed with medium A reagent and permeabilized with medium B reagent (Nordic Mubio) according to manufacturer’s instructions. Cytokines determination was performed by intracellular cytokine staining (ICS) with anti-IFN-γ-APCR700 (BD) and anti-TNF-α-FITC (Miltenyi) monoclonal antibodies and analyzed by flow cytometry using LSR FORTESSA BD.

### 2.7 Functional restoration assay

IFN-γ and TNF-α production as well as CD107a surface expression were assessed following overnight stimulation with IL-12 and IL-18 (5 ng/ml each) in the presence or absence of specific TGF-β and p38 inhibitors. The following inhibitors were used: Galunisertib (1 μM), a selective inhibitor of the TGF-β type I receptor (ALK5) kinase; 5Z-7-oxozeaenol (0.1 μM), an irreversible inhibitor of TAK1 kinase; SB203580 (1 μM), a selective inhibitor of p38 MAPK; and SIS3 (2.5 μM), a specific inhibitor of SMAD3 phosphorylation. All inhibitors were diluted in 1x PBS and added to the samples at the beginning of the stimulation period and maintained throughout the incubation.

Results are expressed as the fold change in the frequency of cytokine-producing and CD107a-expressing NK cells in inhibitor-treated samples relative to untreated controls.

### 2.8 TGF-β-mediated conversion of PBMCs and LINKs

PBMCs from 5 healthy donors and LINKs from 5 HCC patients were thawed and cultured for 7 days under the following conditions: 5×105 cells were seeded per well in 96-well plates in RPMI 1640 medium supplemented with 8% human serum and IL-15 (10 ng/mL; Sigma-Aldrich), in the presence or absence of both recombinant human TGF-β1 (2 ng/mL; PeproTech) and 1 mM 5-azacytidine (Aza; Sigma-Aldrich), as previously described (4). After 3 days of culture, the medium was replaced with fresh medium under the same conditions. On day 7, cells were counted and subsequently seeded overnight in RPMI 1640 medium supplemented with 10% FBS, at 5×10^5^ cells per tube for flow cytometry. During the overnight incubation, cells were divided into different experimental conditions: an unstimulated control and a stimulated condition with IL-12 and IL-18 (5 ng/mL each; both from R&D Systems/Bio-Techne). The following day the IL-12/IL-18–stimulated cells were further subdivided into two groups, either cultured alone or co-cultured with K562 target cells at a 5:1 effector-to-target (E:T) ratio.

Anti-CD107a-PE-Cy7 antibody together with GolgiPlug and GolgiStop (BD) were added to all experimental conditions. Cells were then incubated at 37 °C for 4h before proceeding with the intracellular staining protocol for granzyme B and perforin.

### 2.9 Cytotoxic granules expression

For intracellular detection of cytotoxic granule proteins, cells were first stained with a live/dead viability dye to exclude non-viable cells. Surface staining was then performed using: anti-CD3-BUV805, anti-CD56-APC-R700, anti-CD16-FITC, anti-CD103-BV421, anti-CD49a-RB705 and anti-CD9-BUV395 (BD Biosciences). Following surface staining, cells were washed twice with PBS 2% FBS, then fixed and permeabilized using the BD Cytofix/Cytoperm™ Fixation/Permeabilization Kit (BD) according to the manufacturer’s instructions and incubated with anti-granzyme B-BV510 (BD) and anti-perforin A-AF647 (BD) diluted in Perm/Wash™ buffer for 30 min at 4 °C in the dark. Cells were subsequently washed twice with Perm/Wash buffer, resuspended in staining buffer, and acquired on an LSR FORTESSA BD flow cytometer. Data were analyzed using FACSDiva and FlowJo software.

### 2.10 Cytotoxic potential of TGF-β-stimulated PBMCs

NK cell–mediated cytotoxicity against K562 target cells was assessed by flow cytometry. K562 cells were labeled with 0.5 µM carboxyfluorescein succinimidyl ester (CFSE; Thermo Fisher Scientific) and seeded at 10000 cells per tube for the assay. PBMCs, after 7 days of culture and overnight stimulation as described above, were stained with anti-CD56 and anti-CD3 antibodies (BD), followed by washing and staining with the viability dye 7-AAD (BD). This staining was performed to determine the percentage of NK cells in the PBMC population, enabling adjustment of effector cell numbers to achieve a 5:1 effector-to-target (E:T) ratio. K562 cells cultured without effector cells were used as a negative control to determine the spontaneous death rate of target cells. After a 4h co-incubation at 37 °C, cells were collected, stained with 7-AAD, and analyzed by flow cytometry. Cytotoxicity was calculated as the difference between the percentage of CFSE^+^/7-AAD^+^ K562 cells in experimental and control samples.

### 2.11 Statistical analysis

Statistical analyses were performed using the R statistical software (The R Project for Statistical Computing). Data distribution was assessed using the Kolmogorov–Smirnov test. For paired comparisons, Wilcoxon matched-pairs signed-rank test was applied. For multiple group comparisons, Kruskal–Wallis test followed by Benjamini–Hochberg (BH) correction for multiple testing was used. A two-tailed p-value < 0.05 was considered statistically significant.

## 3 Results

### 3.1 TGF-β Pathway dynamics in NK Cells from tumor and liver compartments

**Figure 1:**
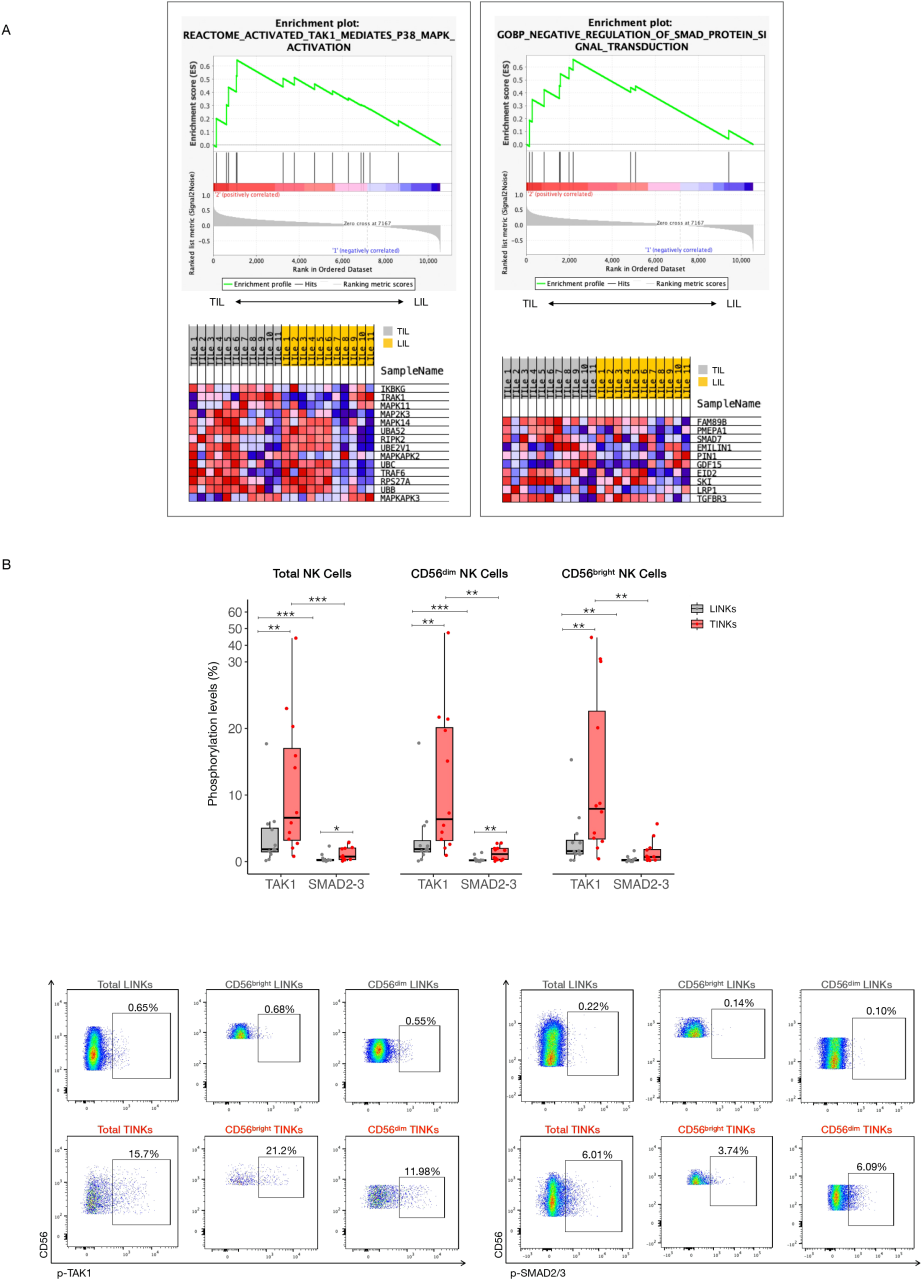
TGF-β Pathway dynamics in NK Cells from tumor and liver compartments. **(A)** GSEA results are presented as enrichment plots and heatmaps of core enrichment genes, highlighting gene sets significantly upregulated in the tumor compartment compared to the liver (TILs, tumor-infiltrating lymphocytes; LILs, liver-infiltrating lymphocytes) (p<0.05, FDR<0.25). **(B)** Phosphorylation levels of SMAD2–3 complex (canonical TGF-β pathway) and TAK1 (non-canonical TGF-β pathway) in Total, CD56^dim^ and CD56^bright^ NK Cells. Below, representative dot plots showing phosphorylation levels of SMAD2–3 complex and TAK1 in Total, CD56^dim^ and CD56^bright^ Liver-infiltrating and Tumor-infiltrating NK Cells (LINKs, TINKs). P-values were calculated by paired Wilcoxon signed-rank test (p=* ≤ 0.05; ** ≤ 0.01; ***≤ 0.001).

Targeted Gene Set Enrichment Analysis (GSEA), performed on 25 curated gene sets associated with canonical (SMADs-dependent) and non-canonical (TAK1-dependent) TGF-β signaling pathways, demonstrated significant upregulation of two key pathways in Tumor-infiltrating NK Cells (TINKs) compared to Liver-infiltrating NK Cells (LINKs):

- REACTOME_ACTIVATED_TAK1_MEDIATES_P38_MAPK_ACTIVATION (NES 1.68, NOM p-val 0.004, FDR q-val 0.086)
- GOBP_NEGATIVE_REGULATION_OF_SMAD_PROTEIN_SIGNAL_TRANSDUCTION (NES 1.59, NOM p-val 0.013, FDR q-val 0.109) (**Figure 1A**).

Core enrichment genes within these upregulated pathways included IKBKG, IRAK1, MAPK11, MAP2K3, and MAPK14 in the TAK1/p38 MAPK pathway, as well as FAM89B, PMEPA1, SMAD7, EMILIN1, PIN1, GDF15, and EID2 in the SMAD regulatory network.

The concurrent upregulation of genes involved in TAK1/p38 MAPK signaling, alongside the suppression of SMAD-dependent pathways, suggests a functional shift in TGF-β signaling dynamics within TINK cells. This shift appears to favor the activation of non-canonical, pro-inflammatory, and stress-associated pathways over canonical SMAD-mediated transcriptional regulation. Notably, the increased expression of SMAD7, PMEPA1, and GDF15—established inhibitors of canonical TGF-β signaling—further supports this regulatory transition.

To validate these findings, the activation of the TGF-β pathway in infiltrating NK cells was assessed by analyzing the phosphorylation levels of SMAD2–3 and TAK1 using flow cytometry. Phosphorylation of SMAD2–3 complex was used as a marker for activation of the canonical SMAD-dependent pathway, while TAK1 phosphorylation indicated engagement of the non-canonical TRAF4/6-TAK1-dependent signaling cascade.

In total NK cells, as well as in both CD56^bright^ and CD56^dim^ subsets, non-canonical TGF-β signaling was significantly upregulated in the tumor compartment compared to the liver, as evidenced by increased TAK1 phosphorylation levels.

Furthermore, a significant increase in phosphorylation of the SMAD2–3 complex was observed in both total NK cells and the CD56^dim^ subset within the tumor, relative to the liver (**Figure 1B**).

Comparative analysis of TGF-β pathway activation across both compartments consistently revealed a preferential activation of non-canonical over canonical signaling in NK cells. This trend was particularly pronounced within the tumor microenvironment, underscoring a context-dependent modulation of TGF-β signaling in infiltrating NK cells (**Figure 1B**).

Overall, these observations might indicate a complex interplay within the tumor microenvironment. Even though GSEA results indicate an enrichment of pathways that inhibit canonical signaling, the canonical SMADs-dependent pathway demonstrates increased phosphorylation in the tumor microenvironment compared to the liver. This can be clarified by examining the relative magnitudes of protein activation: the non-canonical pathway exhibits significantly greater phosphorylation than the canonical pathway in the tumor, directly supporting the GSEA findings. Even with lower activation than the TAK1-dependent pathway, the SMADs pathway shouldn’t be entirely dismissed. Its subtle contribution might not be the main driver, but SMADs signaling could still be crucial in shaping the overall immune environment within the tumor.

### 3.2 TGF-β-driven phenotypic plasticity of NK Cells in tumor microenvironment

**Figure 2:**
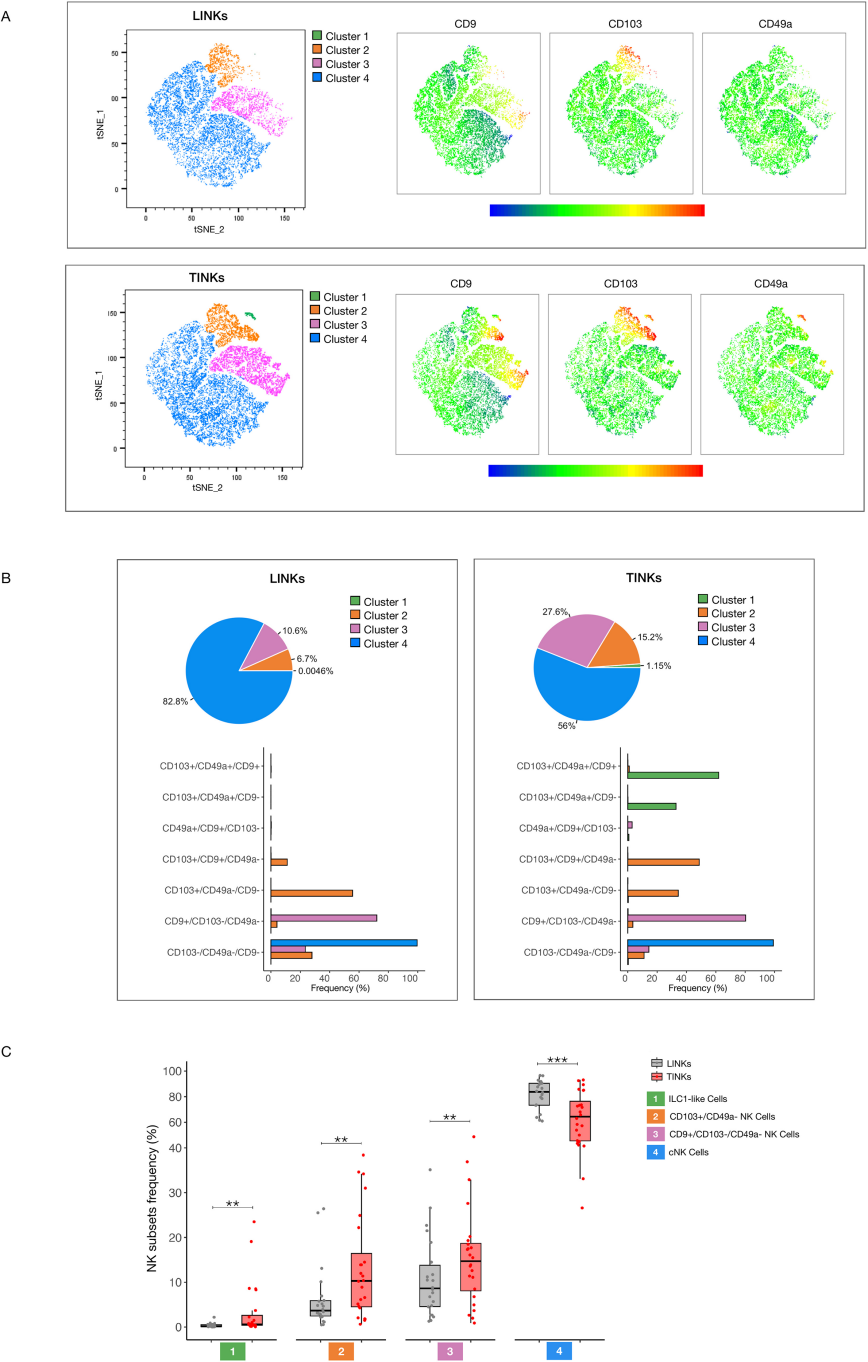
TGF-β mediated plasticity of NK Cells. **(A)** t-SNE analysis of total LINKs (top) and TINKs (bottom). On the right panels, expression levels of CD9, CD103, and CD49a are shown. **(B)** Pie charts show the prevalence of t-SNE-identified clusters (panel A) within total LINKs and TINKs. Frequency distribution of LINK and TINK subpopulations is presented below for each cluster. **(C)** Percentage of ILC1-like (CD103^+^/CD49a^+^), CD103^+^/CD49a^−^, CD9^+^/CD103^−^/CD49a^−^, and cNK (CD103^−^/CD49a^−^/CD9^−^) cells in liver and tumor compartments. P-values were calculated by paired Wilcoxon signed-rank test (p= ** ≤ 0.01; ***≤ 0.001).

To characterize the expression patterns of CD103, CD49a, and CD9 within our patient cohort, we conducted t-distributed Stochastic Neighbor Embedding (t-SNE) analysis, enabling visualization of NK cell distribution across liver and tumor compartments. t-SNE projections revealed a striking enrichment of CD103, CD49a, and CD9 expression in TINKs compared to LINKs, suggesting distinct phenotypic reprogramming within the tumor milieu (**Figure 2A**). Statistical analyses corroborated these findings, demonstrating significantly higher expression levels of these markers in TINKs (**Supplementary Figure 1A**). The gating strategy employed for identifying positive subsets for these markers is detailed in **Supplementary Figure 1B**.

Clustering analysis of t-SNE identified four distinct NK cell subsets. Cluster 1 was almost exclusively restricted to the tumor compartment, though it represented a minor fraction of the total NK population (1.15% in TINKs vs. 0.004% in LINKs). This cluster was predominantly composed of CD103^+^/CD49a^+^/CD9^+^ NK cells (62%), alongside CD103^+^/CD49a^+^/CD9^−^ NK cells (32.9%), populations typically associated with an ILC1-like phenotype (**Figure 2B**).

Cluster 2 exhibited a heterogeneous composition within TINKs, primarily consisting of CD103^+^/CD9^+^/CD49a^−^ NK cells (48.7%) and CD103^+^/CD49a^−^/CD9^−^ NK cells (34.3%), alongside a minor subset of triple-negative conventional NK cells (cNK, 11%). The distribution of Cluster 2 in LINKs displayed similar heterogeneity but with a distinct subset hierarchy: CD103^+^/CD49a^−^/CD9^−^ NK cells were most abundant (55.6%), followed by cNKs (27%) and CD103^+^/CD9^+^/CD49a^−^ NK cells (11.1%) (**Figure 2B**).

Clusters 3 and 4 displayed similar phenotypic distributions across liver and tumor compartments. Cluster 3 was enriched in CD9^+^/CD103^−^/CD49a^−^ NK cells (72.1% in LINKs, 80.3% in TINKs), whereas Cluster 4 consisted almost exclusively of cNK cells (99.6% in LINKs, 99.2% in TINKs) (**Figure 2B**).

Collectively, these findings define distinct NK cell subsets within liver and tumor compartments. Cluster 1 was enriched in ILC1-like NK cells (CD103^+^/CD49a^+^), Cluster 2 was composed of CD103^+^ NK cells lacking CD49a, Cluster 3 was characterized by CD9^+^/CD103^−^/CD49a^−^ NK cells and Cluster 4 was highly enriched in cNK cells.

**Figure 2C** highlights a significant increase in ILC1-like, CD103^+^/CD49a^−^, and CD9^+^/CD103^−^/CD49a^−^ cells within the tumor microenvironment, accompanied by a substantial depletion of cNK cells compared to the liver. Interestingly, in tumor compartment ILC1-like and CD103^+^/CD49a^−^ cells where predominantly represented by CD56^bright^ while cNK by CD56^dim^ NK Cells (**Supplementary Figure 1C**).

To further characterize these subsets, we examined the expression of immune checkpoints PD-1, TIM-3, and TIGIT (**Supplementary Figure 2**). Among the subsets, only cNK cells—similarly to total NK cells—showed increased PD-1 expression in the tumor compartment compared to the liver. Regarding TIM-3, all subsets exhibited higher expression levels in the tumor. Analysis of PD-1 and TIM-3 co-expression revealed a significant enrichment in the tumor for cNK cells and the CD9^+^CD103^−^CD49a^−^ subset, a pattern also observed in total NK cells. In contrast, TIGIT expression followed an opposite trend, with reduced levels in the tumor compared to the liver in both ILC1-like and cNK cells, as well as in total NK cells (**Supplementary Figure 2A**). However, TIGIT was significantly more expressed on TIM-3 positive and PD-1/TIM-3 positive NK cells compared to the corresponding negative populations, in agreement with the inhibitory role of this molecule (**Supplementary Figure 2B**).

Within the tumor compartment, ILC1-like NK cells displayed the highest expression of PD-1, PD-1/TIM-3 co-expression, and TIGIT among all subsets, suggesting that this population may represent a uniquely dysfunctional or highly regulated NK cell state (**Supplementary Figure 2C**).

### 3.3 Subset-specific hierarchies in TGF-β pathway activation among tumor-infiltrating NK cells

**Figure 3:**
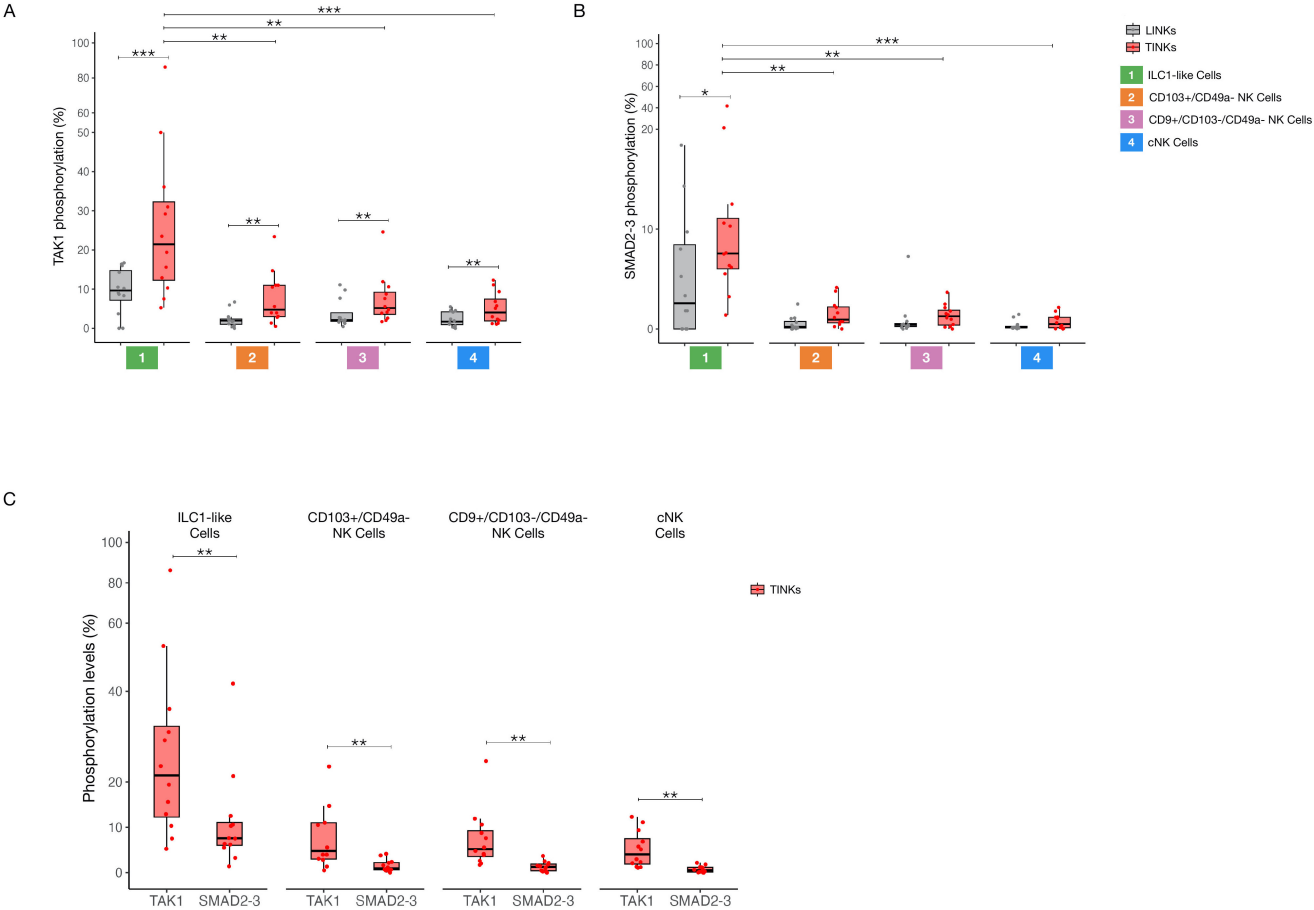
Subset-specific hierarchies in TGF-β pathway activation among infiltrating NK Cells. Phosphorylation levels of TAK1 (non-canonical TGF-β pathway) **(A)** and SMAD 2-3 (canonical TGF-β pathway) **(B)** in liver- and tumor-infiltrating NK subpopulations. **(C)** Comparison of canonical and non-canonical TGF-β pathways activation within tumor-infiltrating NK subsets. P-values were calculated by paired Wilcoxon signed-rank test or with Kruskal–Wallis test followed by Benjamini–Hochberg (BH) correction for multiple comparisons (p=* ≤ 0.05; ** ≤ 0.01; ***≤ 0.001).

We evaluated the activation of both canonical and non-canonical TGFβ signaling pathways across four distinct clusters identified through tSNE analysis. TAK1 phosphorylation was significantly elevated in all tumor-infiltrating NK cell subsets compared to their liver counterparts (**Figure 3A**). Moreover, SMAD2–3 phosphorylation was significantly increased in ILC1-like cells compared to the liver compartment (**Figure 3B**). Notably, tumor-infiltrating ILC1-like cells exhibited the most robust activation of both TGFβ pathways, with a markedly stronger activation of the non-canonical pathway relative to the other TINK subsets (**Figure 3C**).

Additionally, our investigation into the phosphorylation status of SMAD2–3 and TAK1 across various cellular subsets revealed a distinct regulatory mechanism in tumor-infiltrating ILC1-like cells.

In most analyzed populations—both within the liver and tumor microenvironments—SMAD2–3 and TAK1 activation appears to be mutually exclusive (**Supplementary Figure 3**). This conclusion is supported by two key observations: the lack of significant differences between the frequency of single-positive cells and the total positive populations for each marker, and the consistently low frequency of SMAD2-3/TAK1 double-positive cells. This pattern suggests that these signaling pathways operate independently in these cellular contexts.

However, a notable exception was identified within the tumor-infiltrating ILC1-like cell subset. Here, we found that the percentage of cells positive for SMAD2–3 alone was significantly lower than the total population of SMAD2-3-positive cells. This disparity is attributed to a higher prevalence of SMAD2-3/TAK1 double-positive cells in this specific population. In contrast, the frequency of cells positive for TAK1 alone did not differ significantly from the total TAK1-positive population, despite the increased co-activation (**Supplementary Figure 3**).

This differential pattern of activation reveals a potential pathway crosstalk in tumor-infiltrating ILC1-like cells. Our data suggest that while TAK1 activation remains largely an exclusive event, a substantial portion of SMAD2–3 phosphorylation in these cells is dependent on or occurs concurrently with TAK1 activation.

### 3.4 Reduced TGF-βR1 expression in tumor-infiltrating NK cells

**Figure 4:**
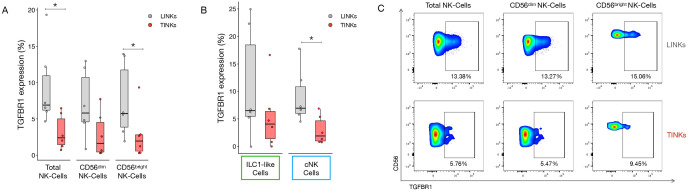
TGF-βR1 expression in liver- and tumor-infiltrating NK cells. **(A)** TGF-βR1 expression in Total, CD56^dim^, CD56^bright^ LINKs and TINKs. **(B)** TGF-βR1 expression in liver- and tumor-infiltrating ILC1-like and cNK cells. **(C)** Representative dot plots showing TGF-βR1 expression in Total, CD56^dim^, CD56^bright^ LINKs and TINKs. P-values were calculated by paired Wilcoxon signed-rank test (p=* ≤ 0.05).

We evaluated TGF-βR1 expression in LINKs and TINKs. Both total NK cells and the CD56^bright^ subset displayed significantly lower TGF-βR1 expression in the tumor compartment compared to the liver (**Figure 4A**). This pattern was consistent across NK cell subsets, cNK cells infiltrating the tumor showed significantly reduced receptor expression relative to their liver counterparts (**Figure 4B**) Although not reaching statistical significance, CD56^dim^ and ILC1-like cells exhibited a similar trend, with tumor-infiltrating cells exhibiting lower TGF-βR1 expression than their liver counterparts. **Figure 4C** shows representative dot plots of TGF-βR1 expression in both LINKs and TINKs.

### 3.5 The impaired functionality of cNK cells can be restored by inhibiting TGF-β signaling

**Figure 5:**
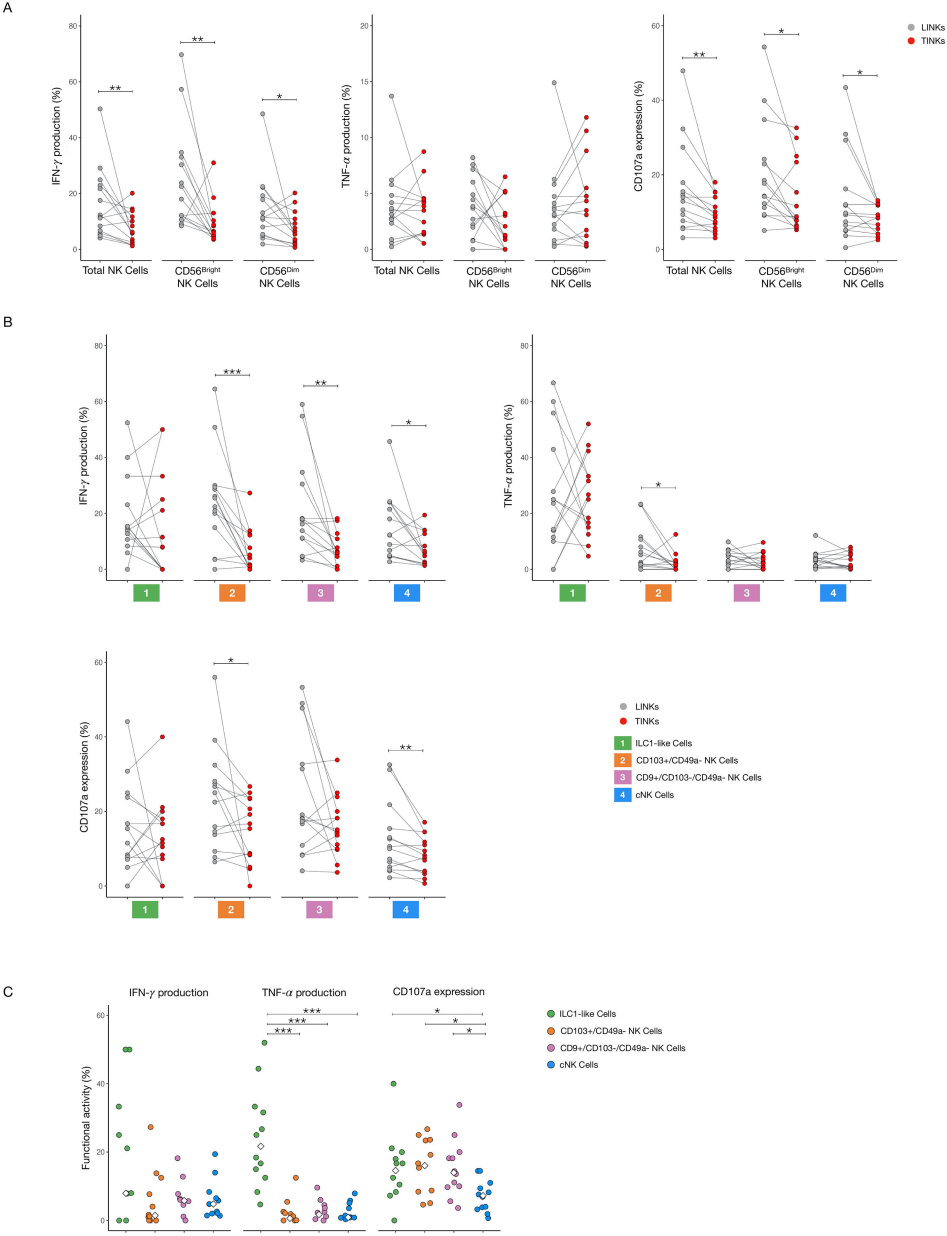
Functional analysis of liver- and tumor-infiltrating NK Cells. IFN-γ and TNF-α production, CD107a expression in Total, CD56^bright^ and CD56^dim^ NK cells **(A)** and in ILC1-like, CD103^+^/CD49a^−^, CD9^+^/CD103^−^/CD49^−^ and cNK cells **(B)** after overnight stimulation with IL-12 and IL-18. **(C)** Comparison of cytokines production and CD107a expression within tumor-infiltrating subpopulations. The median values are indicated by a white rhombus symbol. P-values were calculated by paired Wilcoxon signed-rank test or with Kruskal–Wallis test followed by Benjamini–Hochberg (BH) correction for multiple comparisons (p=* ≤ 0.05; ** ≤ 0.01; ***≤ 0.001).

To evaluate NK cell functionality, we measured IFN-γ and TNF-α production along with degranulation capacity (as indicated by CD107a expression) in LINKs and TINKs cells. Total TINKs, as well as the CD56^bright^ and CD56^dim^ subsets, exhibited significantly reduced functionality, with lower IFN-γ production and CD107a expression compared to LINKs (**Figure 5A**). This pattern of reduced functional activity was consistent across various NK cell subsets— including CD103^+^/CD49a^-^, CD9^+^/CD103^-^/CD49a^-^, and cNK cells—with the notable exception of tumor-infiltrating ILC1-like cells, which maintained functional capacity similar to that of their liver counterparts (**Figure 5B**).

Within the tumor microenvironment, cNK cells showed the lowest CD107a expression, indicating impaired degranulation, while ILC1-like cells produced substantial amounts of TNF-α, a pro-inflammatory cytokine that has been associated with tumor progression and poor prognosis in HCC (**Figure 5C**) (18).

**Figure 6:**
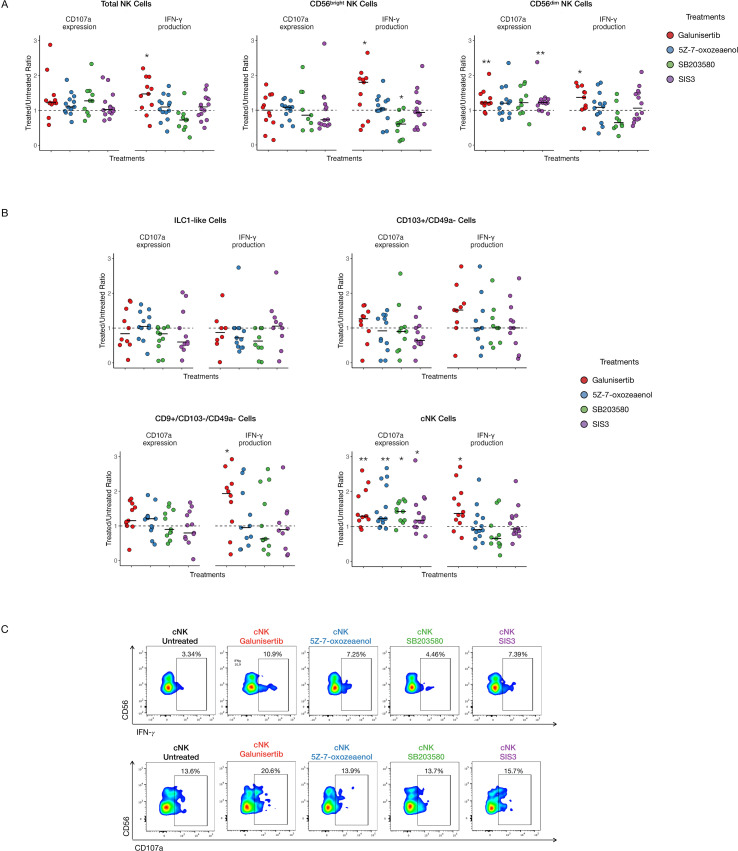
Functional restoration of tumor-infiltrating NK Cells. IFN-γ production and CD107a expression in Total, CD56^bright^ and CD56^dim^ NK cells **(A)** and in ILC1-like, CD103^+^/CD49a^−^, CD9^+^/CD103^−^/CD49^−^ and cNK cell subsets **(B)** after overnight treatment and stimulation with IL-12 and IL-18. All graphs show the ratio of the treated condition to the untreated control. The median value for each treatment is indicated by a black line. **(C)** Representative dot plots showing IFN-γ production and CD107a expression in untreated and treated conditions in TINKs. P-values were calculated using the Wilcoxon signed-rank test (comparing to a hypothetical median of 1) and corrected for multiple comparisons using the Benjamini–Hochberg (BH) method (p=* ≤ 0.05; ** ≤ 0.01).

To explore potential therapeutic strategies targeting TGF-β signaling, NK cells from both compartments were treated overnight with Galunisertib (TGF-β type I receptor kinase inhibitor), SB203580 (p38 MAPK inhibitor), 5Z-7-oxozeaenol (TAK1 kinase inhibitor), and SIS3 (inhibitor of SMAD3 phosphorylation). Galunisertib significantly enhanced IFN-γ production in TINKs, across total, CD56^bright^, and CD56^dim^ subsets. Within the CD56^dim^ TINK population, both Galunisertib and SIS3 markedly increased CD107a expression, indicating enhanced degranulation capacity (**Figure 6A**).

Analysis of TINK subsets identified through t-SNE revealed that only cNK cells responded to all treatments with improved degranulation capacity. Moreover, Galunisertib significantly boosted IFN-γ production for cNK and for CD9^+^/CD103^-^/CD49a^-^ subsets and close to statistical significance for CD103^+^/CD49a^-^ subset (**Figure 6B**). Representative dot plots in **Figure 6C** illustrate IFN-γ production and CD107a expression in tumor-infiltrating cNKs for both untreated and treated samples.

The only inhibitory effect observed for our treatments was for SB203580 on IFN-γ production in CD56^bright^ cells, potentially reflecting a dose-dependent toxic effect of the drug on IFN-γ production, in contrast to CD107a expression, which could be enhanced in cNK cells.

Interestingly, none of the treatments significantly affected the functional profile of LINK cells (**Supplementary Figure 4**). Moreover, TNF-α production remained unaltered in all conditions (data not shown).

The Kruskal-Wallis test, followed by Benjamini-Hochberg correction for multiple comparisons, revealed no significant differences in the expression of CD103, CD49a, and CD9 across the different condition groups (p > 0.05) and cell viability between the different conditions [untreated 77.3 (IQR 14.8), 5Z-7-oxozeaenol 77.1 (IQR 13.4), SIS3 80.6 (IQR 8.95), SB203580 85.0 (IQR 17.5), Galunisertib 80.0 (IQR 11.9)] (data not shown).

### 3.6 CD107a and cytotoxic granules expression in LINKs and TINKs

To gain a deeper understanding of LINKs and TINKs cytotoxicity, we analyzed the expression of the degranulation marker CD107a after stimulation with K562 target cells. Additionally, we quantified the intracellular expression of the cytotoxic granules perforin A and granzyme B.

**Figure 7:**
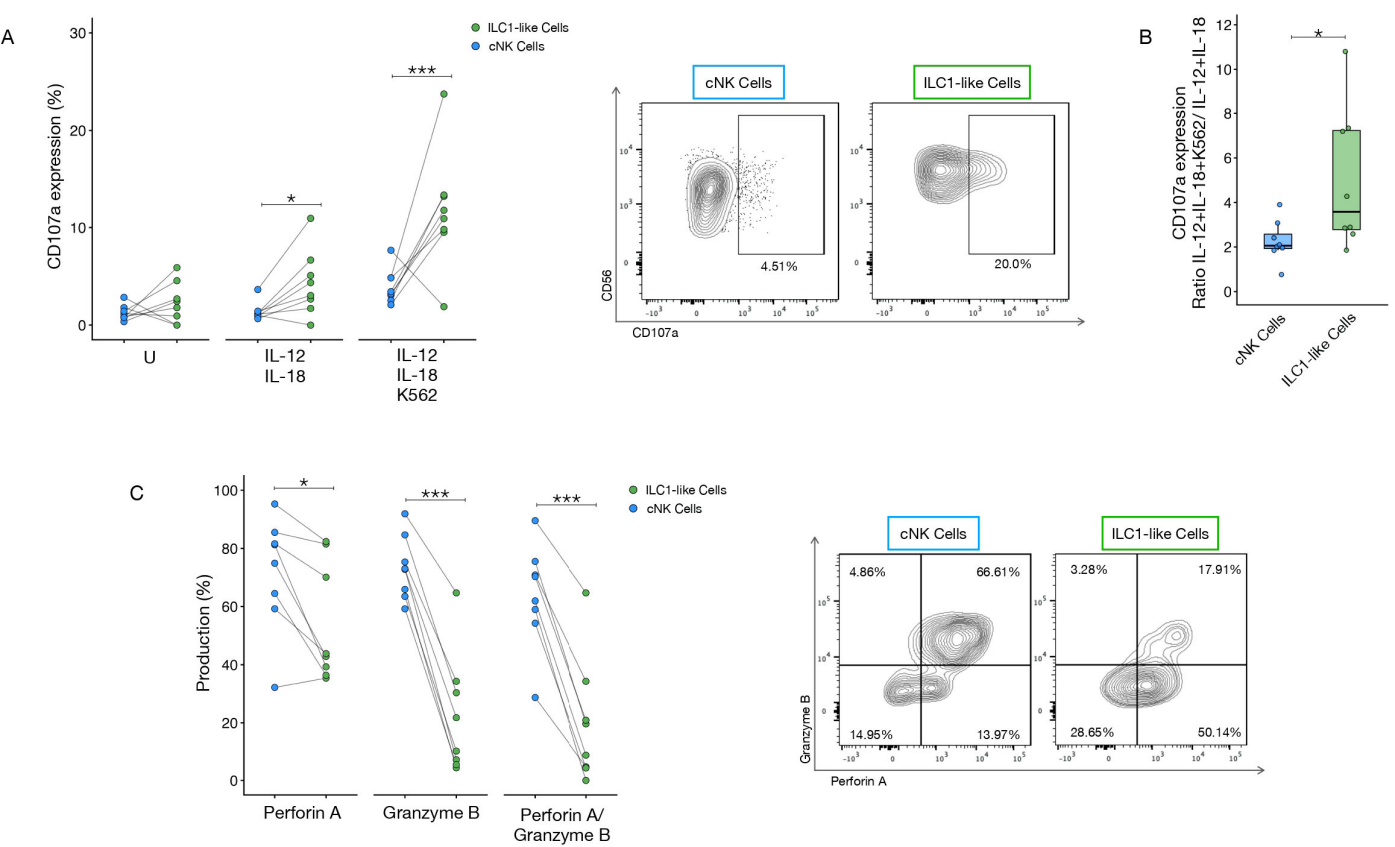
CD107a and cytotoxic granules expression in tumor-infiltrating ILC1-like and cNK cells. **(A)** CD107a expression in ILC1-like and cNK cells in unstimulated (U), stimulated with IL-12 and IL-18 (5ng/ml each) and stimulated with IL-12, IL-18 and K562 conditions. **(B)** CD107a expression evaluated as IL-12/IL-18 + K562 and IL-12/IL-18 ratio of cNK and ILC1-like cells. **(C)** Perforin A, granzyme B expression and perforin A/granzyme B co-expression in ILC1-like and cNK cells. P-values were calculated by paired Wilcoxon signed-rank test (p=* ≤ 0.05; ***≤ 0.001).

In the total NK cell population, stimulation with IL-12 and IL-18 induced a modest, but non-significant, increase in CD107a expression in the liver compartment compared to the tumor, with a higher expression with K562 target cells (**Supplementary Figure 5A**). Upon subsets analysis, we observed that ILC1-like cells’ CD107a expression was significantly lower in the tumor microenvironment compared to the liver when the cells were stimulated with IL-12/IL-18 (**Supplementary Figure 5A**). Interestingly, tumor infiltrating-ILC1-like cells exhibited significantly higher CD107a expression compared to cNK cells derived from the same compartment, especially upon stimulation with K562 target cells (**Figure 7A**). As shown in **Figure 7B**, the expression ratio, calculated as the degranulation induced by IL-12 and IL-18 stimulation relative to the combined effect with K562 target cells, was markedly elevated in the ILC1-like cell population. This finding complements an already lower basal expression of CD107a in cNK cells, indicating that not only ILC1-like cells possess a greater expression of CD107a, but they also demonstrate a distinctly more potent overall responsiveness compared to their cNK cell counterparts. We next assessed the cytotoxic granule content of LINKs and TINKs. Total NK cells showed no significant differences in granzyme B and perforin A expression between the tumor and liver compartments, though a trend of higher perforin A was noted in the tumor-infiltrating cells (**Supplementary Figure 5B**). Significant differences in the NK subsets were confined to the cNK cells, which exhibited a significant increase in both single granzyme B expression and perforin A/granzyme B double-positivity in the tumor-infiltrating cells compared to those in the liver. Conversely, ILC1-like cells only showed a trend toward reduced cytotoxic granule expression in the tumor compared to the liver (**Supplementary Figure 5B**). When comparing NK cell subsets within the tumor compartment, we observed that ILC1-like cells displayed significantly lower levels of perforin A and granzyme B expression compared to cNK cells (**Figure 7C**). The proportion of cells co-expressing both perforin A and granzyme B was also markedly reduced in ILC1-like cells compared with cNK cells (**Figure 7C**).

### 3.7 TGF-β-mediated conversion of PBMCs and LINKs

**Figure 8:**
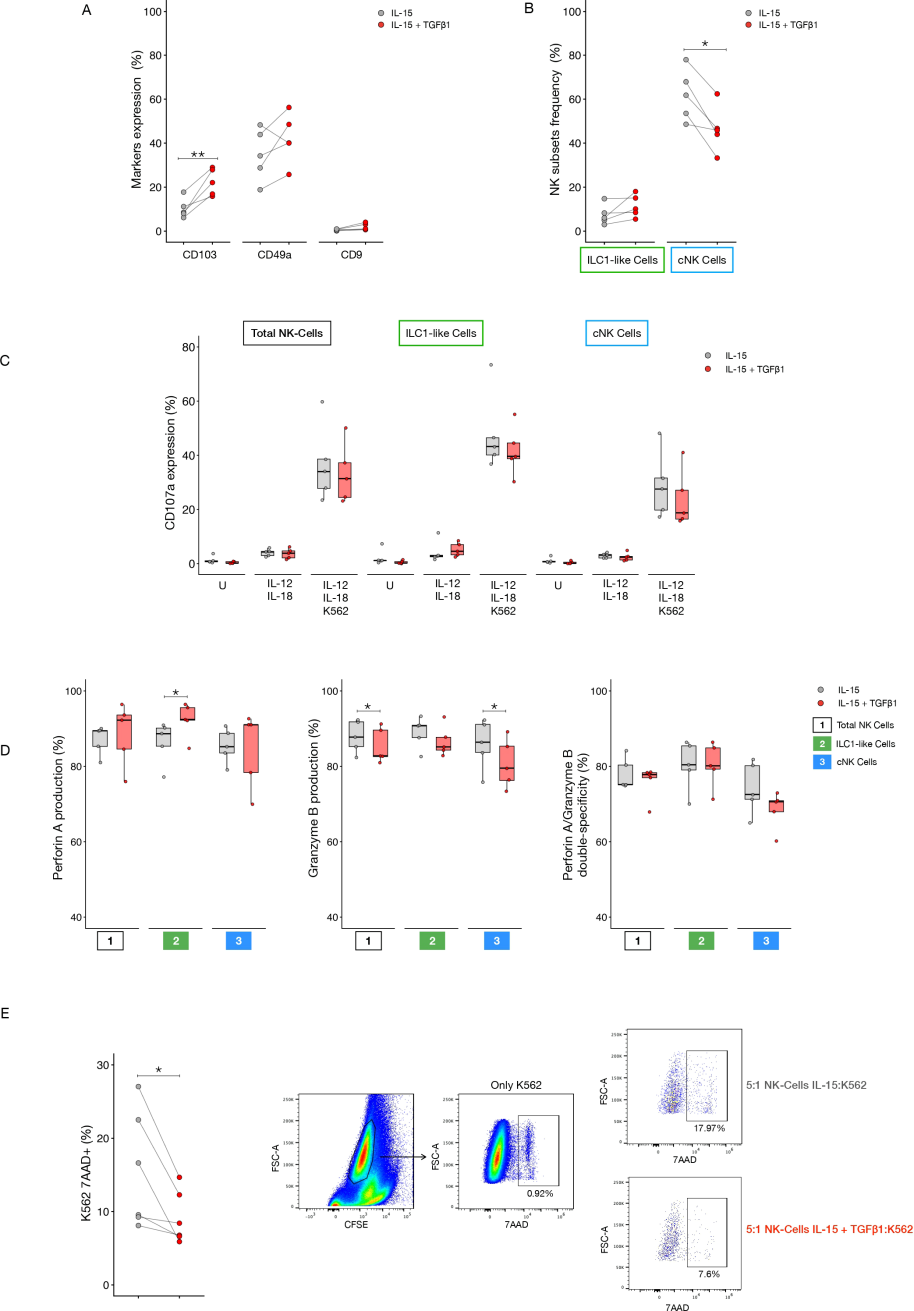
TGF-β-mediated conversion of PBMCs from healthy donors. All the parameters and assays were assessed and performed after 7 days of culture with RPMI 8% human serum supplemented with IL15 (10ng/ml) and in the presence or absence of TGF-β. **(A)** CD103, CD49a and CD9 expression in NK cells in the two culture conditions. **(B)** Frequency of ILC1-like and cNK cells in the two different culture conditions. **(C)** CD107a expression in Total, ILC1-like and cNK cells in unstimulated, stimulated with IL12 and IL18 (5ng/ml each) and stimulated with IL12, IL18 and K562 conditions. **(D)** Perforin A, granzyme B expression and perforin A/granzyme B co-expression in Total, ILC1-like and cNK cells. **(E)** Cytotoxic NK cells potential against K562. K562 were labeled with CFSE and seeded at a 5:1 effector to target ratio (NK:K562 ratio). After a 4h incubation cells were stained with 7-AAD to determine their death rate. Representative dot plots on the right show spontaneous death rate of K562 cells alone and death rate of the target cells after co-culture with NK cells coming from the control condition (IL-15) and the TGF-β stimulated condition. P-values were calculated by paired Wilcoxon signed-rank test (p=* ≤ 0.05; ** ≤ 0.01).

To validate the role of TGF-β in driving the induction of an ILC1-like phenotype, we exposed PBMCs from healthy donors and LINKs to TGF-β for 7 days. Following the 7 day culture, we first evaluated the expression of key surface markers (CD103, CD49a, and CD9) and NK cell subset frequencies. TGF-β stimulation of PBMCs resulted in a significant increase in CD103 expression and a significant reduction in cNK cell frequency compared to the control condition (IL-15 alone) (**Figures 8A, B**). While not reaching statistical significance, the expression of the individual markers CD49a and CD9 were both elevated in the TGF-β-stimulated condition (**Figure 8A**). Concordantly, the frequency of ILC1-like cells showed an increasing trend under the same TGF-β stimulation (**Figure 8B**).

We next assessed the cytotoxic potential of the cultured PBMCs. No significant differences were observed in CD107a expression between the two conditions, though a trend of decreased CD107a was evident in the TGF-β-stimulated cells (**Figure 8C**). However, significant differences were observed in cytotoxic granule expression (**Figure 8D**). Perforin A expression was significantly higher in the ILC1-like cells stimulated with TGF-β compared to the control condition. Conversely, granzyme B expression was reduced in the Total NK and cNK cell populations upon TGF-β stimulation relative to the IL-15 control.

Finally, to directly evaluate the cytotoxic potential of these phenotypically altered cells, we performed a co-culture killing assay using K562 target cells. As shown in **Figure 8E**, the PBMCs stimulated with TGF-β exhibited a significantly reduced cytotoxic capacity compared to the control cells.

**Figure 9:**
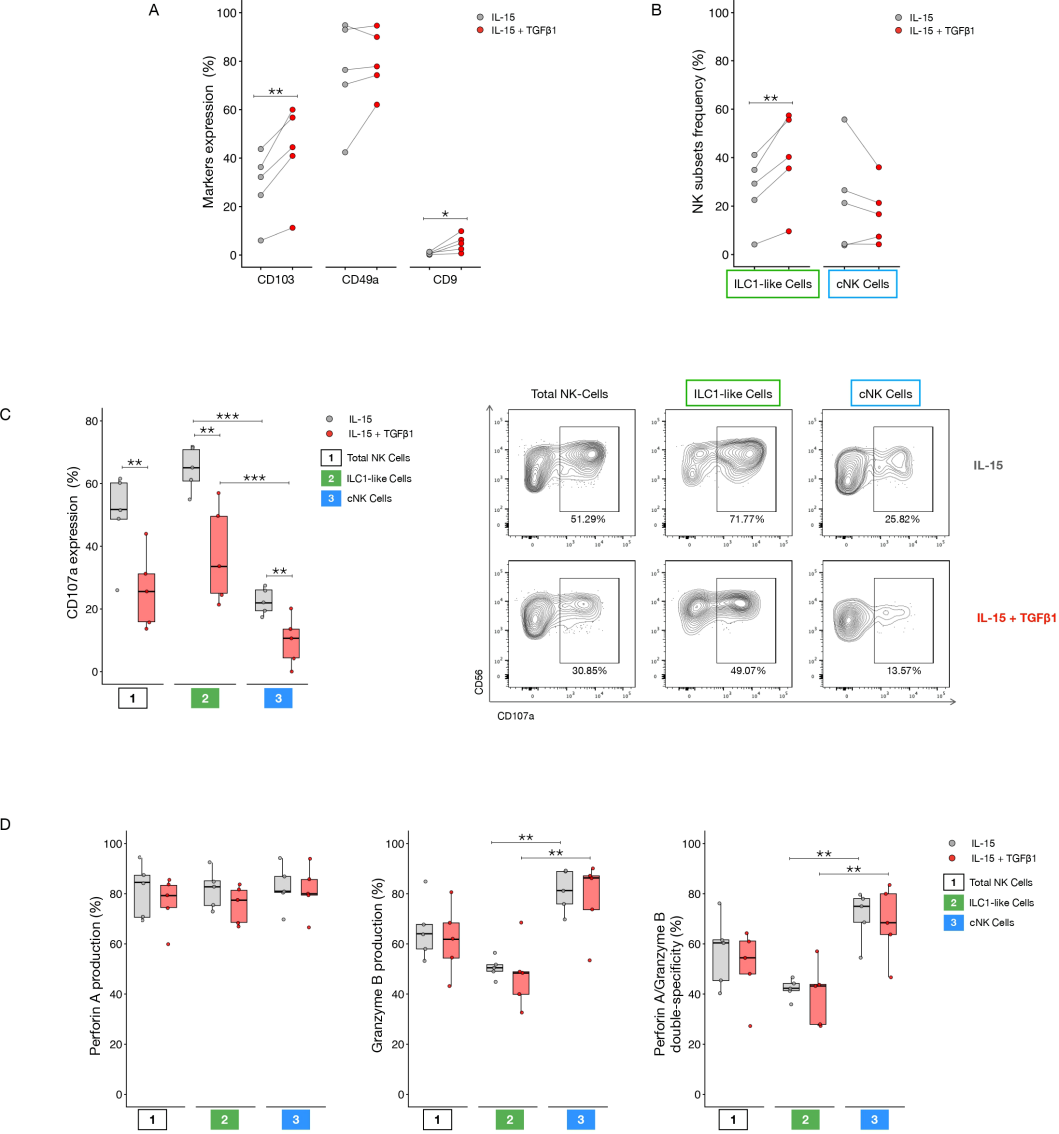
TGF-β-mediated conversion of Liver-infiltrating NK cells. All the parameters and assays were assessed and performed after 7 days of culture with RPMI 8% human serum supplemented with IL15 (10ng/ml) and in the presence or absence of TGF-β. **(A)** CD103, CD49a and CD9 expression in LINKs in the two culture conditions. **(B)** Frequency of ILC1-like and cNK cells in the two different culture conditions. **(C)** CD107a expression in Total NK, ILC1-like and cNK cells after stimulation with IL12, IL18 and K562. **(D)** Perforin A, granzyme B expression and perforin A/granzyme B co-expression in Total NK, ILC1-like and cNK cells. P-values were calculated by paired Wilcoxon signed-rank test (p= ** ≤ 0.01; ***≤ 0.001).

Similarly, exposure of LINKs to TGF-β resulted in a significant increase in the expression of CD103 and CD9, along with a significant increase in the frequency of ILC1-like cells, when compared to the control condition (**Figures 9A, B**). While the frequency of cNK cells did not show a significant difference between the two conditions, there was a trend toward a reduced frequency of these cells in the TGF-β-stimulated condition (**Figure 9B**). In contrast to the PBMCs, 7 days of TGF-β stimulation led to a significantly reduced CD107a expression in LINKs across total NK, ILC1-like, and cNK populations when cultured with K562 target cells (**Figure 9C**). Consistent with observations in TINKs shown previously, the ILC1-like cells displayed a significantly higher CD107a expression compared to the cNK cells, both in the control and the TGF-β-stimulated conditions (**Figure 9C**). Conversely, regarding the expression of cytotoxic granules (**Figure 9D**), we observed a higher expression of single granzyme B and perforin A/granzyme B double-positivity in the cNK cells compared to the ILC1-like cells, in both the control and TGF-β-stimulated conditions, consistent with what we observed in TINKs. In summary, the exposure of both PBMCs and LINKs to TGF-β resulted in a concurrent ILC1-like phenotypic shift and a profound cytotoxic impairment, strongly supporting the notion that this microenvironmental factor is pivotal in creating the dysfunctional state found in tumor-infiltrating NK cells.

## 4 Discussion

Our findings reveal a distinct modulation of TGF-β signaling dynamics in NK cells infiltrating the tumor microenvironment of HCC patients. Through both transcriptomic and functional analyses, we demonstrate that TINKs, preferentially engage the non-canonical TGF-β pathway, characterized by TAK1/p38 MAPK activation, rather than the canonical SMAD-dependent signaling route. Despite this preference, we detected higher SMAD2–3 phosphorylation in both total and CD56^dim^ NK cell subsets compared to their liver-resident counterparts, indicating concurrent, albeit weaker, intra-tumoral engagement of the canonical pathway. The enhanced downstream signaling activity in Tumor-Infiltrating NK cells occurs despite a downregulation of the TGF-β receptor 1 (TGF-βR1) expression when compared to liver-resident NK cells. This apparent contradiction can be explained as a cellular adaptation to the high concentrations of TGF-β1 present in the tumor microenvironment. We propose that the reduced expression of TGF-βR1 represents a compensatory downregulation mechanism, whereby TINKs attempt to mitigate the overwhelming inhibitory signal, as previously demonstrated (18). However, the potent concentration of TGF-β1 is sufficient to continuously drive the phosphorylation of SMAD2–3 and the activation of the non-canonical TAK1/p38 MAPK pathway, thereby contributing to functional anergy and exhausted phenotype of these cells. Supporting a TGF-β–driven phenotypic shift, we identified within the tumor a population of ILC1-like NK cells, characterized by co-expression of CD103 and CD49a, almost absent in the liver. Although representing a minor fraction of the total TINK population, this subset was significantly enriched compared to the liver, consistent with previous models where sustained non-canonical TGF-β signaling drives the conversion of NK cells toward an ILC1-like phenotype. Consistent with this, ILC1-like NK cells exhibited the highest levels of activation for both TGF-β signaling arms, with predominant engagement of the non-canonical pathway. The relatively low frequency of ILC1-like cells in our cohort could reflect the early-stage disease status of the HCC patients analyzed. It is plausible that in more advanced tumors, prolonged TGF-β exposure may further expand this population.

To validate the role of TGFβ in driving the induction of an ILC1-like phenotype, we exposed PBMCs from healthy donors and LINKs to TGF-β1 for 7 days. In both settings, this resulted in a cellular profile that closely resembled that of tumor-infiltrating cells, confirming the ability of TGF-β to drive this phenotypic change.

Functionally, ILC1-like NK cells exhibited a higher capacity to secrete cytokines, particularly TNF-α, a cytokine known to promote tumor progression in HCC due to its proinflammatory and proangiogenic properties (19). Furthermore, while these cells showed higher expression of the degranulation marker CD107a compared to cNK cells, this was associated with a diminished capacity to produce the key cytotoxic molecules granzyme B and perforin A. This profile suggests that while the degranulation machinery is engaged, the cells lack the necessary effector proteins to execute lytic function. This finding is consistent with the known distribution of these subsets, as the ILC1-like cells are associated with the CD56^bright^ compartment, while conventional NK cells predominantly belong to the cytotoxic CD56^dim^ subset, the primary producers of granzyme B and perforin A.

This pattern in ILC1-like cells may indicate that these cells are not merely anergic but have been functionally rewired to favor pro-tumorigenic inflammatory pathways over direct anti-tumor cytotoxicity. Phenotypic analysis further supported this state, as ILC1-like NK cells expressed the highest levels of multiple inhibitory immune checkpoints, including PD-1, TIM-3, and TIGIT, when compared to other NK cell subsets. This expression profile may reflect chronic stimulation within the tumor microenvironment and could suggest a state of heightened immunoregulatory potential, possibly shaped by TGF-β signaling.

Tumor-infiltrating cNK cells exhibited a distinct form of functional impairment compared to ILC1-like cells. Despite being characterized by a robust production of granzyme B and perforin A, they showed reduced expression of the degranulation marker CD107a and diminished cytokine production.

Interestingly, while residing in the same tumor microenvironment as ILC1-like cells, cNK subset exhibited TGF-β-induced functional impairment rather than upregulation of tissue-residency markers, such as CD103 and CD49a. This different response likely reflects the predominance of terminally differentiated, less plastic CD56^dim^ cells over the CD56^bright^ subset within the cNK population. As previously demonstrated, TGF-β may be responsible of NKG2D, NKp30, NKp46, and DNAM-1 downregulation on this subset with inhibition of cytotoxic response (8, 20).

Pharmacological inhibition of TGF-β signaling partially restored NK cell function, with the most pronounced effects observed in tumor-derived cNKs. All inhibitors tested—Galunisertib, SB203580, 5Z-7-oxozeaenol, and SIS3—enhanced CD107a expression in these cells, and Galunisertib also significantly increased IFN-γ production. These results suggest that even in cNK subsets where canonical signaling appears less active, SMAD3 inhibition can confer functional benefits.

Moreover, treatment with SIS3, a specific SMAD3 inhibitor, significantly improved CD107a expression in CD56^dim^ tumor-infiltrating NK cells (TINKs)—a subset where we observed increased SMAD2–3 phosphorylation compared to their liver-resident counterparts. Given that conventional NK (cNK) cells predominantly fall within the CD56^dim^ compartment, it is plausible that with a larger sample size, a similar trend in canonical pathway activation may emerge as statistically significant specifically within cNKs, a subset that also showed a positive response to SIS3 treatment.

While cNKs exhibit lower TAK1/SMAD activation compared to ILC1-like cells, this activation is nonetheless heightened in the tumor microenvironment. This distinction is crucial, as TGF-β pathway inhibitors, which were ineffective against liver-resident NKs (LINKs), demonstrated a clear impact on tumor-infiltrating cNKs. A modest but biologically relevant engagement of the SMAD pathway—albeit less prominent than non-canonical signaling—could explain the positive effects of SIS3 observed in this population. This contrasts with ILC1-like cells which, despite high TAK1/SMADs activation, maintained their functionality and cytokine production when treated with the same drugs, suggesting a differential susceptibility to TGF-β-mediated suppression.

Collectively, our findings suggest that TGF-β signaling shapes NK cell function in a subset- and context-dependent manner. While non-canonical signaling predominates in TINKs and drives phenotypic reprogramming toward an ILC1-like state, canonical SMADs signaling may still exert functional constraints that can be therapeutically targeted. The partial restoration of NK cell function following TGF-β pathway inhibition highlights the potential of this approach to reverse tumor-induced dysfunction and bolster innate immune responses in HCC. However, our data also underscore the need for caution: the immunological consequences of modulating TGF-β are highly context-specific and may vary according to disease stage and NK cell subset composition.

In conclusion, in this study, we demonstrate prevalent activation of the non-canonical TGF-β pathway in tumor-infiltrating NK cells, which may drive the plastic differentiation of functional ILC1-like cells beside being responsible of functional impairment of different NK-cell subsets. Future studies in longitudinal and advanced-stage HCC cohorts will be essential to define the optimal therapeutic window and to disentangle the dualistic role of TGF-β in maintaining tissue homeostasis versus mediating immune suppression. In this context, combinatorial strategies that incorporate TGF-β blockade with current first-line therapies may hold promise in reprogramming the TME to favor anti-tumor immunity.

## Data Availability

The datasets presented in this study can be found in online repositories. The names of the repository/repositories and accession number(s) can be found below: https://www.ncbi.nlm.nih.gov/geo/, GSE183349.
