# Supplementary material for: TGF-β-driven NK Cells plasticity in hepatocellular carcinoma
Source: Front Immunol. 2025 Nov 5;16:1651129. doi: 10.3389/fimmu.2025.1651129 (PMC12627035; doi:10.3389/fimmu.2025.1651129)
Supplement: Supplementary file 1 [file Table1.docx]

Supplementary Table 1. List of 25 curated gene sets associated with both canonical and non-canonical TGF-β signaling pathways used to perform targeted GSEA.

| **TGF-β signaling pathways** |
| --- |
| GOBP_NEGATIVE_REGULATION_OF_SMAD_PROTEIN_SIGNAL_TRANSDUCTION.v2024.1.Hs.gmt |
| GOBP_POSITIVE_REGULATION_OF_SMAD_PROTEIN_SIGNAL_TRANSDUCTION.v2024.1.Hs.gmt |
| GOBP_REGULATION_OF_SMAD_PROTEIN_SIGNAL_TRANSDUCTION.v2024.1.Hs.gmt |
| GOBP_SMAD_PROTEIN_SIGNAL_TRANSDUCTION.v2024.1.Hs.gmt |
| GOCC_SMAD_PROTEIN_COMPLEX.v2024.1.Hs.gmt |
| GOMF_CO_SMAD_BINDING.v2024.1.Hs.gmt |
| GOMF_I_SMAD_BINDING.v2024.1.Hs.gmt |
| GOMF_R_SMAD_BINDING.v2024.1.Hs.gmt |
| GOMF_SMAD_BINDING.v2024.1.Hs.gmt |
| KOINUMA_TARGETS_OF_SMAD2_OR_SMAD3.v2024.1.Hs.gmt |
| REACTOME_TGF_BETA_RECEPTOR_SIGNALING_ACTIVATES_SMADS.v2024.1.Hs.gmt |
| WP_TGFB_SMAD_SIGNALING.v2024.1.Hs.gmt |
| BIOCARTA_P38MAPK_PATHWAY.v2024.1.Hs.gmt |
| KEGG_MAPK_SIGNALING_PATHWAY.v2024.1.Hs.gmt |
| PHONG_TNF_RESPONSE_VIA_P38_COMPLETE.v2024.1.Hs.gmt |
| REACTOME_ACTIVATED_TAK1_MEDIATES_P38_MAPK_ACTIVATION.v2024.1.Hs.gmt |
| REACTOME_IRAK2_MEDIATED_ACTIVATION_OF_TAK1_COMPLEX.v2024.1.Hs.gmt |
| REACTOME_JNK_C_JUN_KINASES_PHOSPHORYLATION_AND_ACTIVATION_MEDIATED_BY_ACTIVATED_HUMAN_TAK1.v2024.1.Hs.gmt |
| REACTOME_TICAM1_TRAF6_DEPENDENT_INDUCTION_OF_TAK1_COMPLEX.v2024.1.Hs.gmt |
| WP_P38_MAPK_SIGNALING.v2024.1.Hs.gmt |
| BIOCARTA_TGFB_PATHWAY.v2024.1.Hs.gmt |
| KEGG_TGF_BETA_SIGNALING_PATHWAY.v2024.1.Hs.gmt |
| REGULATION_OF_TRANSFORMING_GROWTH_FACTOR_BETA_RECEPTOR_SIGNALING_PATHWAY.v2024.1.Hs.gmt |
| TGFB_UP.V1_DN.v2024.1.Hs.gmt |
| TGFB_UP.V1_UP.v2024.1.Hs.gmt |
