# Supplementary figures and images for: TGF-β-driven NK Cells plasticity in hepatocellular carcinoma
Source: Front Immunol. 2025 Nov 5;16:1651129. doi: 10.3389/fimmu.2025.1651129 (PMC12627035; doi:10.3389/fimmu.2025.1651129)

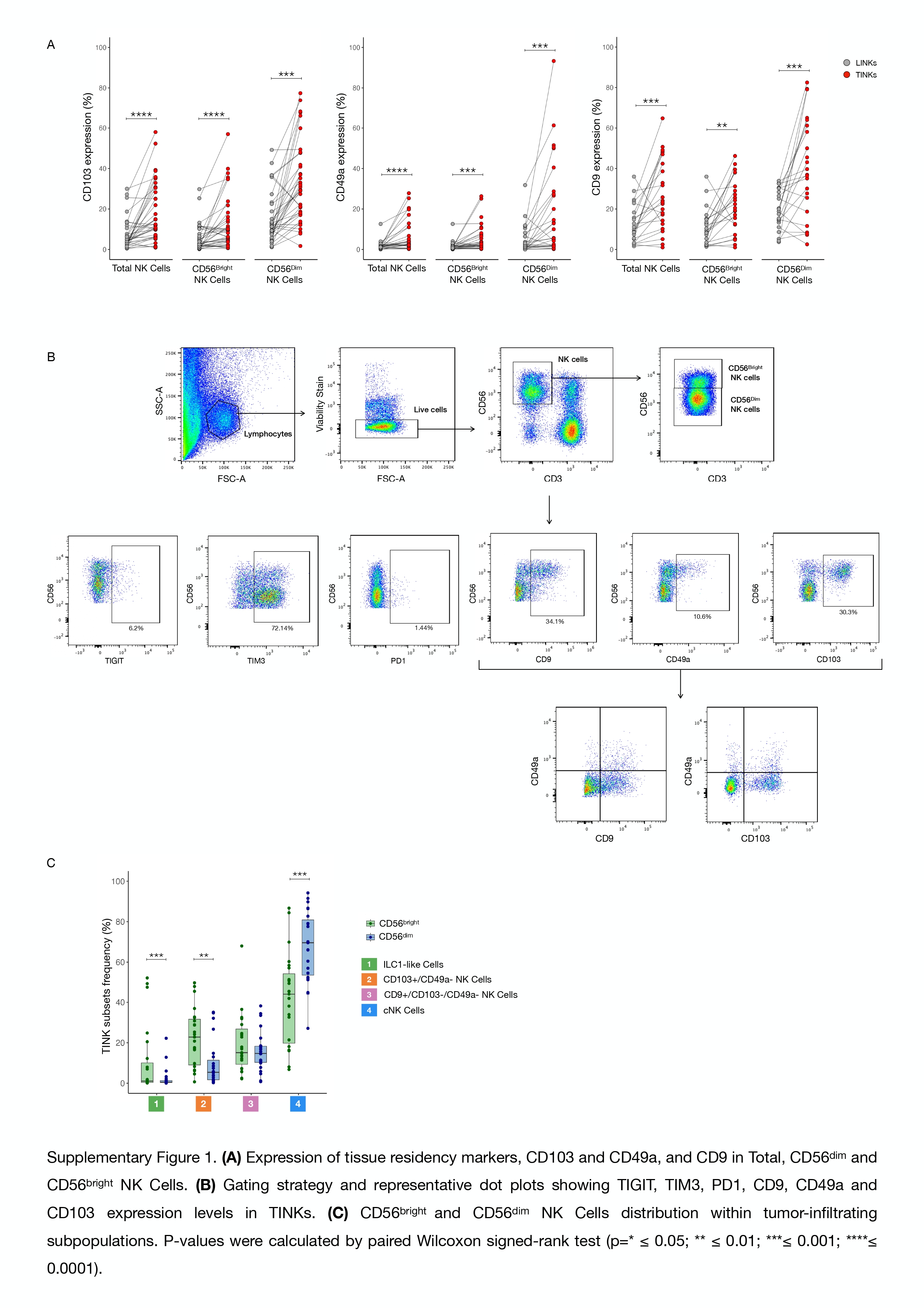

Supplement: Supplementary file 2 [file Image1.jpeg]

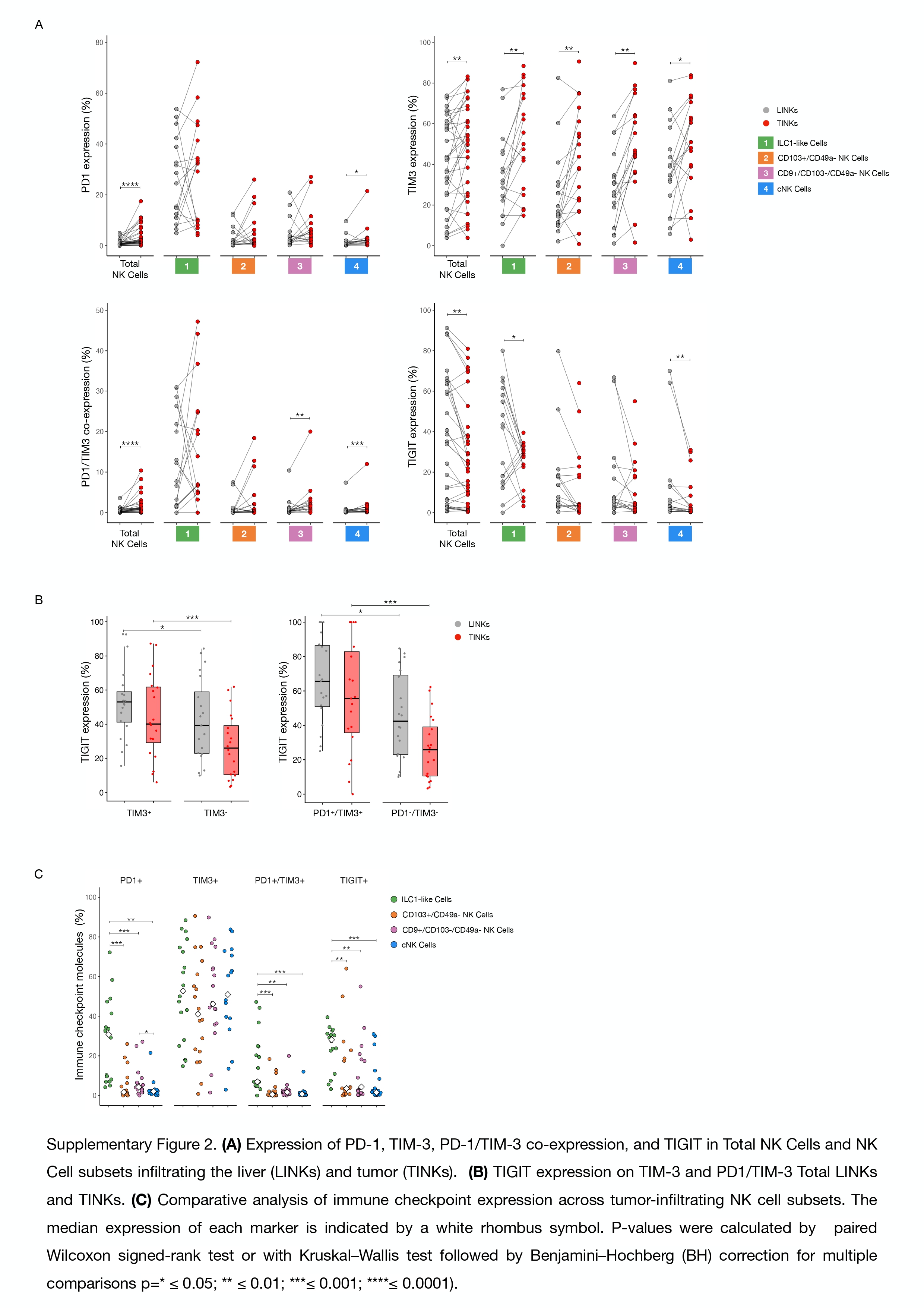

Supplement: Supplementary file 3 [file Image2.jpeg]

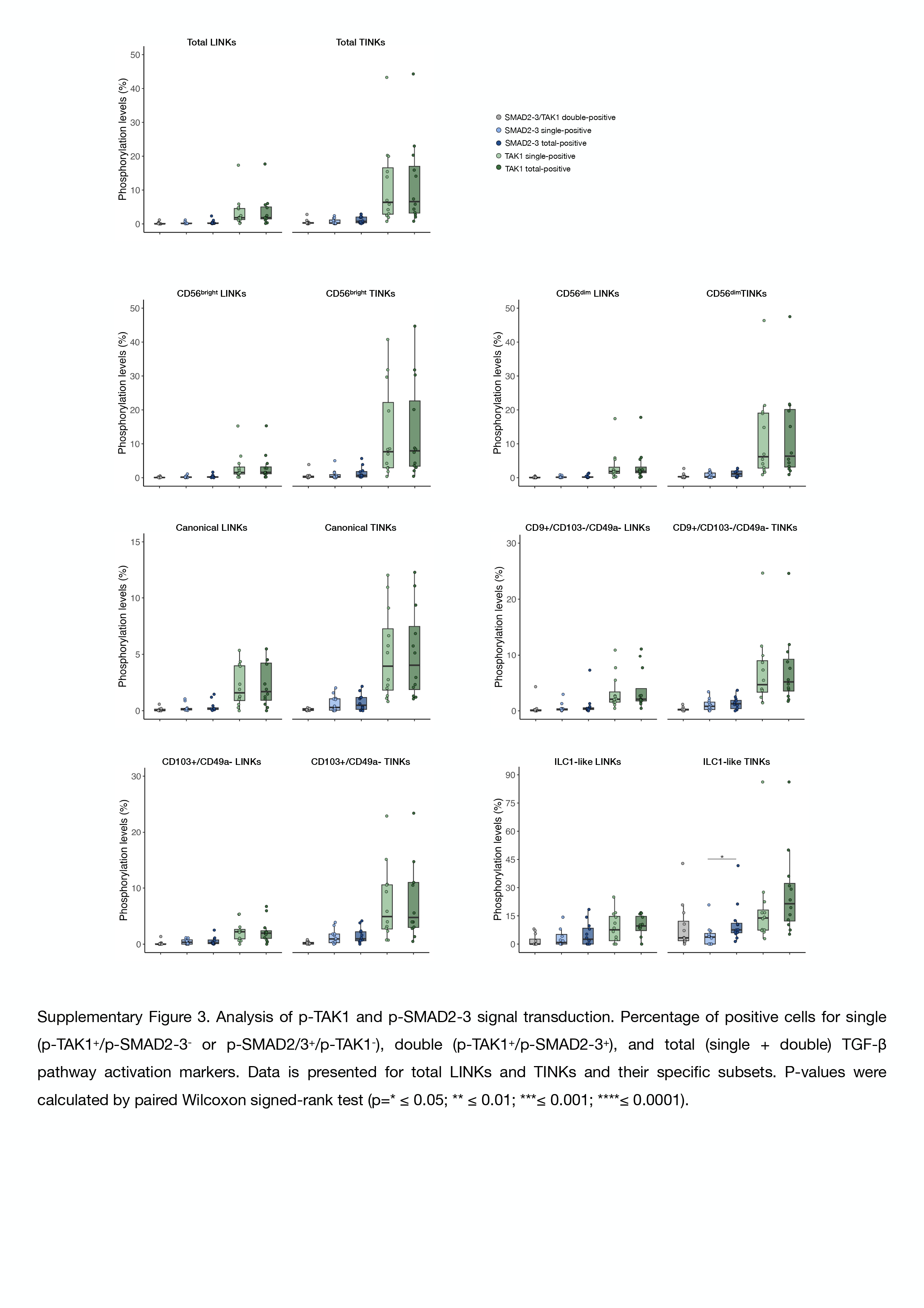

Supplement: Supplementary file 4 [file Image3.jpeg]

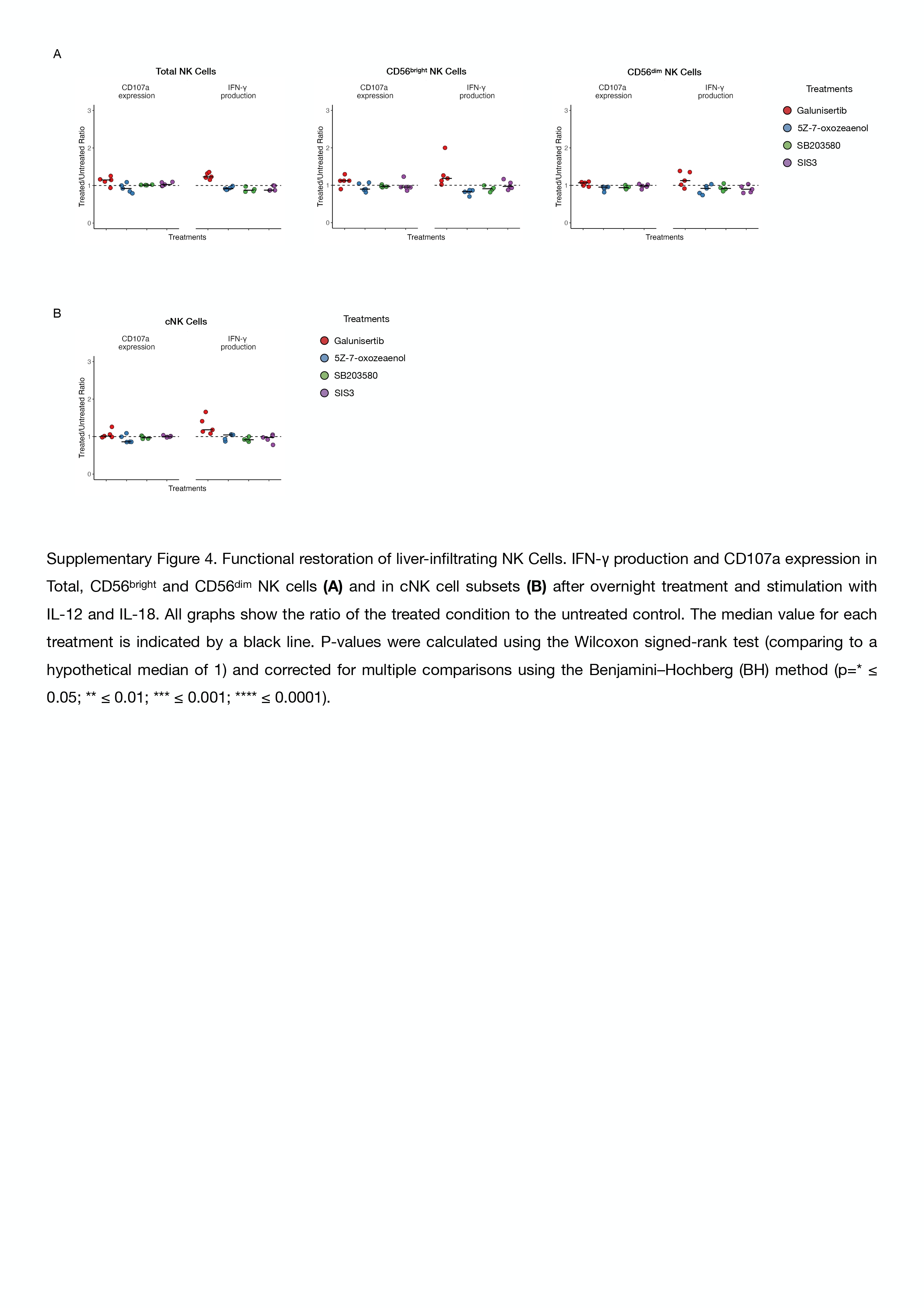

Supplement: Supplementary file 5 [file Image4.jpeg]

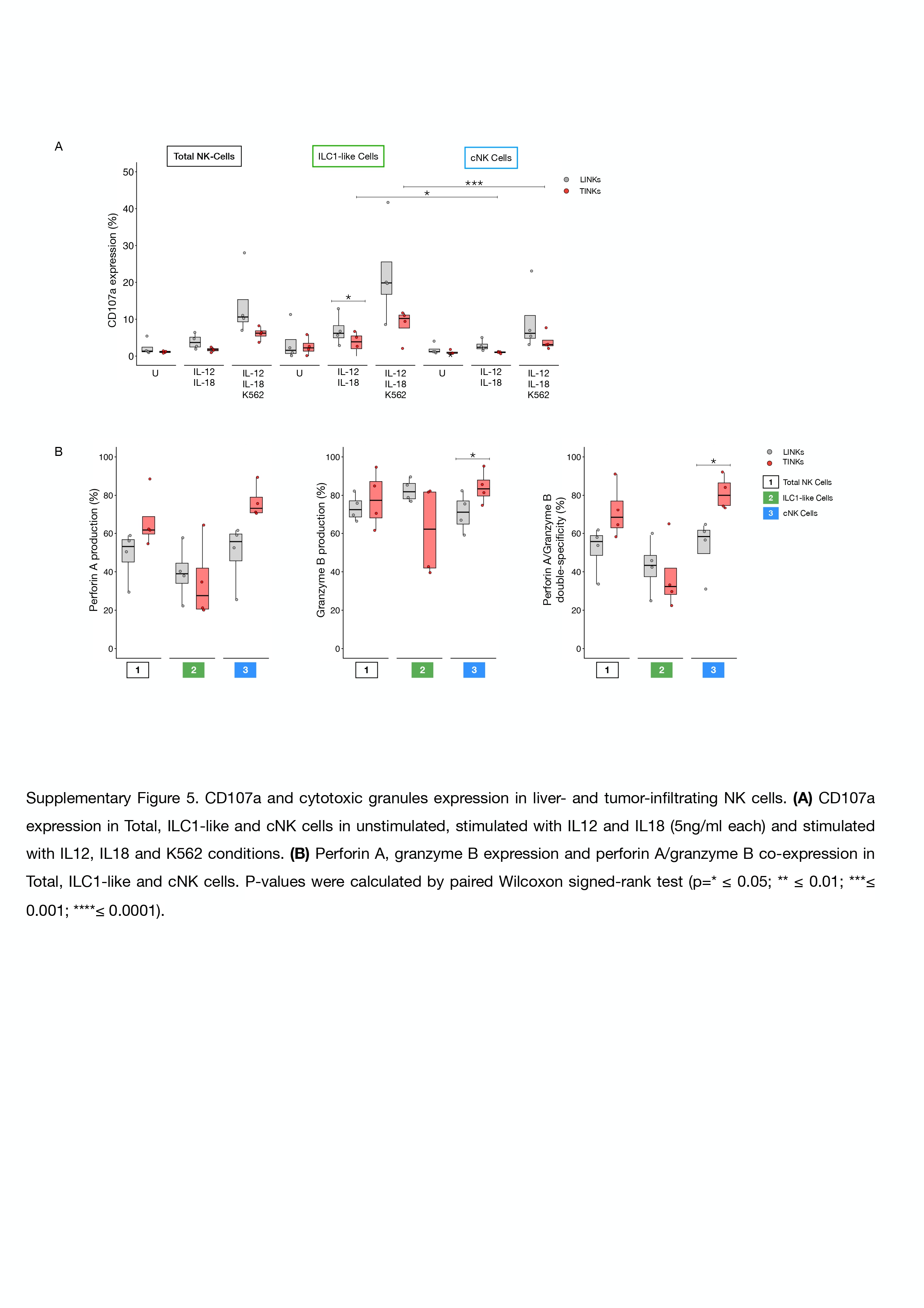

Supplement: Supplementary file 6 [file Image5.jpeg]
